# The Mutational Spectrum in a Cohort of Charcot-Marie-Tooth Disease Type 2 among the Han Chinese in Taiwan

**DOI:** 10.1371/journal.pone.0029393

**Published:** 2011-12-19

**Authors:** Kon-Ping Lin, Bing-Wen Soong, Chih-Chao Yang, Li-Wen Huang, Ming-Hong Chang, I-Hui Lee, Antony Antonellis, Yi-Chung Lee

**Affiliations:** 1 Department of Neurology, National Yang-Ming University School of Medicine, Taipei, Taiwan; 2 The Neurological Institute, Taipei Veterans General Hospital, Taipei, Taiwan; 3 Department of Neurology, National Taiwan University Hospital, Taipei, Taiwan; 4 Section of Neurology, Taichung Veterans General Hospital, Taichung, Taiwan; 5 Institute of Brain Science, National Yang-Ming University School of Medicine, Taipei, Taiwan; 6 Department of Human Genetics and Department of Neurology, University of Michigan Medical School, Ann Arbor, Michigan, United States of America; University Medical Center Groningen, University of Groningen, The Netherlands

## Abstract

**Background:**

Charcot-Marie-Tooth disease type 2 (CMT2) is a clinically and genetically heterogeneous group of inherited axonal neuropathies. The aim of this study was to extensively investigate the mutational spectrum of CMT2 in a cohort of patients of Han Chinese.

**Methodology and Principal Findings:**

Genomic DNA from 36 unrelated Taiwanese CMT2 patients of Han Chinese descent was screened for mutations in the coding regions of the *MFN2, RAB7, TRPV4, GARS, NEFL, HSPB1, MPZ, GDAP1, HSPB8, DNM2, AARS* and *YARS* genes. Ten disparate mutations were identified in 14 patients (38.9% of the cohort), including p.N71Y in *AARS* (2.8%), p.T164A in *HSPB1* (2.8%), and p.[H256R]+[R282H] in *GDAP1* (2.8%) in one patient each, three *NEFL* mutations in six patients (16.7%) and four *MFN2* mutations in five patients (13.9%). The following six mutations were novel: the individual *AARS, HSPB1* and *GDAP1* mutations and c.475-1G>T, p.L233V and p.E744M mutations in *MFN2.* An *in vitro* splicing assay revealed that the *MFN2* c.475-1G>T mutation causes a 4 amino acid deletion (p.T159_Q162del). Despite an extensive survey, the genetic causes of CMT2 remained elusive in the remaining 22 CMT2 patients (61.1%).

**Conclusions and Significance:**

This study illustrates the spectrum of CMT2 mutations in a Taiwanese CMT2 cohort and expands the number of CMT2-associated mutations. The relevance of the *AARS* and *HSPB1* mutations in the pathogenesis of CMT2 is further highlighted. Moreover, the frequency of the *NEFL* mutations in this study cohort was unexpectedly high. Genetic testing for *NEFL* and *MFN2* mutations should, therefore, be the first step in the molecular diagnosis of CMT2 in ethnic Chinese.

## Introduction

Charcot-Marie-Tooth disease (CMT) is a clinically and genetically heterogeneous group of inherited neuropathies sharing common characteristics of progressive distal muscle weakness and atrophy, foot deformities, distal sensory loss and depressed tendon reflexes. They can be further categorized according to the pathological or electrophysiological features, and the inherited axonal neuropathy CMT type 2 (CMT2) is one of the subgroups [1]. To date, mutations in as many as 14 different genes have been implicated in CMT2, including *KIF1B* (CMT2A1) [2], *MFN2* (CMT2A2) [3], *RAB7* (CMT2B) [4], *TRPV4* (CMT2C) [5], *GARS* (CMT2D) [6], *NEFL* (CMT2E) [7], *HSPB1* (CMT2F) [8], *MPZ* (CMT2I/J) [9], [10], *GDAP1* (CMT2K) [11], [12], *HSPB8* (CMT2L) [13], [14], *DNM2* (CMT2M) [15], *AARS* (CMT2N) [16], *LAMIN A* (AR-CMT2A) [17] and *MED25* (AR-CMT2B) [18]. Among them, mutations in *MFN2* have been found in approximately 11–24.2% of CMT2 patients [19]–[22], whereas *AARS* and *TRPV4* mutations were only recently identified in limited CMT2 families [5], [16], and mutations in other genes were found in only a few patients.

Dominant-intermediate CMT (DI-CMT) is a rare form of CMT with an autosomally dominant inheritance and intermediate median nerve conduction velocities of 25–45 m/s. DI-CMT is often misdiagnosed as CMT2, particularly if the diagnosis is based on the examination of a single patient. Three causative genes for DI-CMT have been identified: *DNM2* (DI-CMTB) [23], *YARS* (DI-CMTC) [24] and *MPZ* (DI-CMTD) [25].

A comprehensive knowledge of the mutational spectrum and frequency of mutations within the CMT2 populations is important. In a Taiwanese cohort of CMT2 of Han Chinese origin, we extensively surveyed the mutations in the *MFN2*, *RAB7*, *TRPV4*, *GARS*, *NEFL*, *HSPB1*, *MPZ*, *GDAP1*, *HSPB8*, *DNM2*, *AARS* and *YARS* genes and report here the genetic and clinical features of the mutations we investigated.

## Methods

### Ethics Statement

The protocols for this study were approved by the Institutional Review Board of the Taipei Veterans General Hospital. Written informed consent was obtained from all of the participants.

### Patients

Thirty-six CMT2 families of Han Chinese descent were enrolled in this study. These families were selected from a continuous series of 251 CMT pedigrees ascertained at the neurology clinic of Taipei Veterans General Hospital, Taiwan by standard electrophysiological evaluation. The diagnostic guidelines for CMT2 described in the report of the 2nd Workshop of the European CMT consortium were adopted [26]. Sensory involvement was demonstrated in all the probands of the 36 CMT2 families by nerve conduction studies (NCS) to exclude hereditary motor neuropathy (Table S1). The 17p11.2 duplication or deletion, and mutations in *PMP22*, *CX32* and its promoter, and *TTR* had been excluded first by DNA analysis of the proband in each family. The mode of inheritance was autosomal dominant in 20 familial cases, autosomal recessive in 7, and apparently sporadic in 9 with no evidence of family history of peripheral neuropathy (Table S1).

### Mutation analysis

Genomic DNA was extracted from peripheral blood using standard protocols. Mutational analyses of the *MFN2, RAB7, TRPV4, GARS, NEFL, HSPB1, MPZ, GDAP1, HSPB8, DNM2, AARS* and *YARS* genes were performed by PCR amplification using intronic primers and direct DNA sequencing. The sense and antisense strands of each amplicon were sequenced using the Big Dye 3.1 di-deoxy terminator method (Applied Biosystems, Foster City, CA) and the ABI Prism 3700 Genetic Analyzer (Applied Biosystems). The amplicon sequences were compared against published human gene sequences in the National Center for Biotechnology Information (NCBI) database (http://www.ncbi.nlm.nih.gov) to identify putative mutation. After validation of any sequence variations in both the sense and antisense strands, subcloning and subsequent sequencing of the amplicons were further performed to confirm the sequence changes. Phylogenetic conservation of the mutation sites was analyzed by aligning the amino acid sequences from several species (retrieved from the Entrez protein database in the NCBI database) using the ClustalX 2.012 program [27].

### Analysis of abnormal splicing at *MFN2* c.475-1G>T, the splice acceptor mutation in *MFN2* intron 5, using the minigene method

We analyzed the splicing alteration of the *MFN2* c.475-1G>T mutation using an *in vitro* minigene method as previously described [28]. In brief, we cloned the entire genomic DNA sequence of human beta-globin (*HBB*) into pCDNA3.1 to construct the backbone of the artificial minigene. We then independently cloned a genomic DNA fragment that spanned from the 3′ end of intron 4 to the 5′ end of intron 7 of human *MFN2* that was amplified from a patient with the *MFN2* c.475-1G>T mutation or a normal control (wild type) subject into the *BsrGI* site within intron 2 of *HBB*. The minigene clones harboring either the wild type or mutant splice acceptor sequences were selected and verified by repeated sequencing. The minigenes were transfected into 293T and HeLa cells using Lipofectamine 2000 (Invitrogen, Carlsbad, CA). Forty-eight hours after transfection, total RNA was extracted and reverse-transcribed. The minigene transcripts were amplified using primers corresponding to exons 5 and 7 of *MFN2*. The amplicons were analyzed by electrophoresis and sequenced to ascertain any abnormal splicing at *MFN2* c.475-1G>T.

### Haplotype analysis of the patients carrying the *NEFL* p.E396K mutation

Seven individuals from 3 unrelated families (including 5 patients and 2 unaffected individuals) carrying the *NEFL* p.E396K mutation were haplotyped (Figure S1). A haplotype analysis was performed to explore the possible founder effect of p.E396K using seven polymorphic microsatellite markers flanking the *NEFL* gene: D8S1739, D8S1771, D8S382, and D8S1839 are located telomeric, and D8S1989, D8S481, and D8S1734 are centromeric to *NEFL*. These seven markers cover a region of 4.6 Kosambi centimorgans (KcM, sex-averaged). All of the information regarding the primer sequences and allele marker sizes were obtained from the National Center for Biotechnology Information (NCBI) database.

## Results

**Figure 1:**
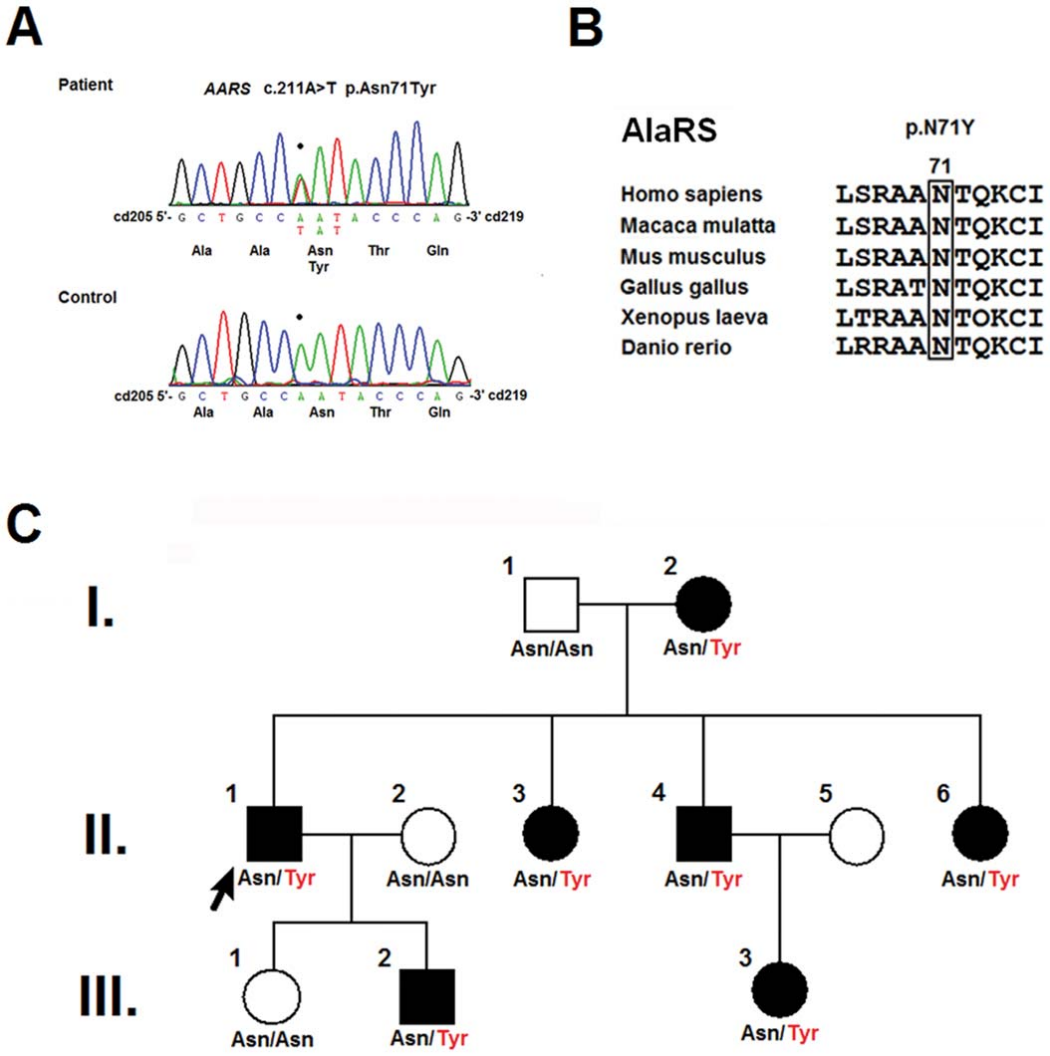
*AARS* p.N71 is evolutionarily conserved, and p.N71Y segregates with CMT2N disease. (A) Sense strand electropherogram of patient and control genomic DNA, showing the heterozygous *AARS* mutation c.211A>T in the patient DNA, corresponding to the amino acid substitution N71Y. (B) Evolutionarily conserved *AARS* p.N71 is shown using a protein sequence alignment of AlaRS orthologs. (C) The pedigree shows segregation of the p.N71Y mutation (the amino acid change shown in red) with CMT2N. The arrow indicates the proband (Patient II-1). The squares and circles represent males and females, and the close and open symbols represent affected and unaffected members. Family member II-5 was not genotyped.

**Figure 2:**
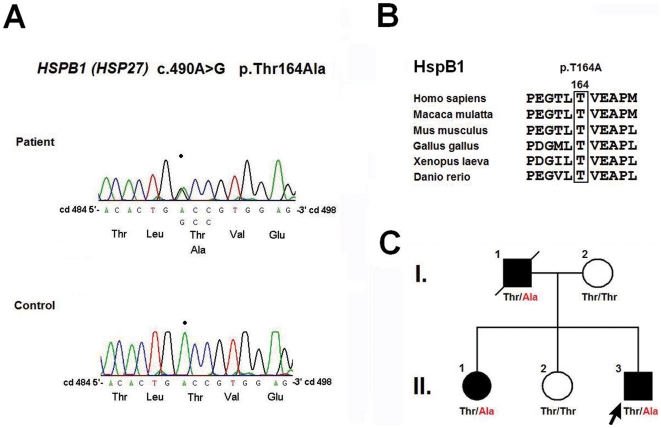
*HSPB1* p.T164 is evolutionarily conserved and segregates with CMT2F in the family. (A) Sense strand electropherogram of patient and control genomic DNA, showing the heterozygous *HSPB1* mutation c.490A>G in the patients DNA, corresponding to the amino acid substitution T164A. (B) Evolutionarily conserved *HSPB1* p.T164 is shown using a protein sequence alignment of HspB1 orthologs. (C) The pedigree depicts segregation of the T164A mutation (the amino acid change shown in red) with CMT2F.

Ten disparate mutations were identified in 14 of the 36 unrelated patients (38.9% of our cohort), including p.N71Y in *AARS* (2.8%), p.T164A in *HSPB1* (2.8%), and a compound heterozygous p.[H256R]+[R282H] mutation in *GDAP1* (2.8%) in one single patient each (Figure 1A, 2A, 3A). *NEFL* mutations were identified in 6 patients (16.7%), including p.E396K, p.P8R and p.P22S in three, two, and one patients each. In addition, *MFN2* mutations were identified in 5 patients (13.9%), including p.R364W in two and c.475-1G>T, p.L233V and p.E744M in one patient each. The genetic, clinical and electrophysiological features for the patients harboring these mutations are summarized in Table 1. No mutations were found in *RAB7, TRPV4, GARS, MPZ, HSPB8, DNM2,* or *YARS* genes. Despite an extensive genetic survey, the genetic causes of CMT2 remain elusive in 22 of the CMT2 patients (61.1%).

**Figure 3:**
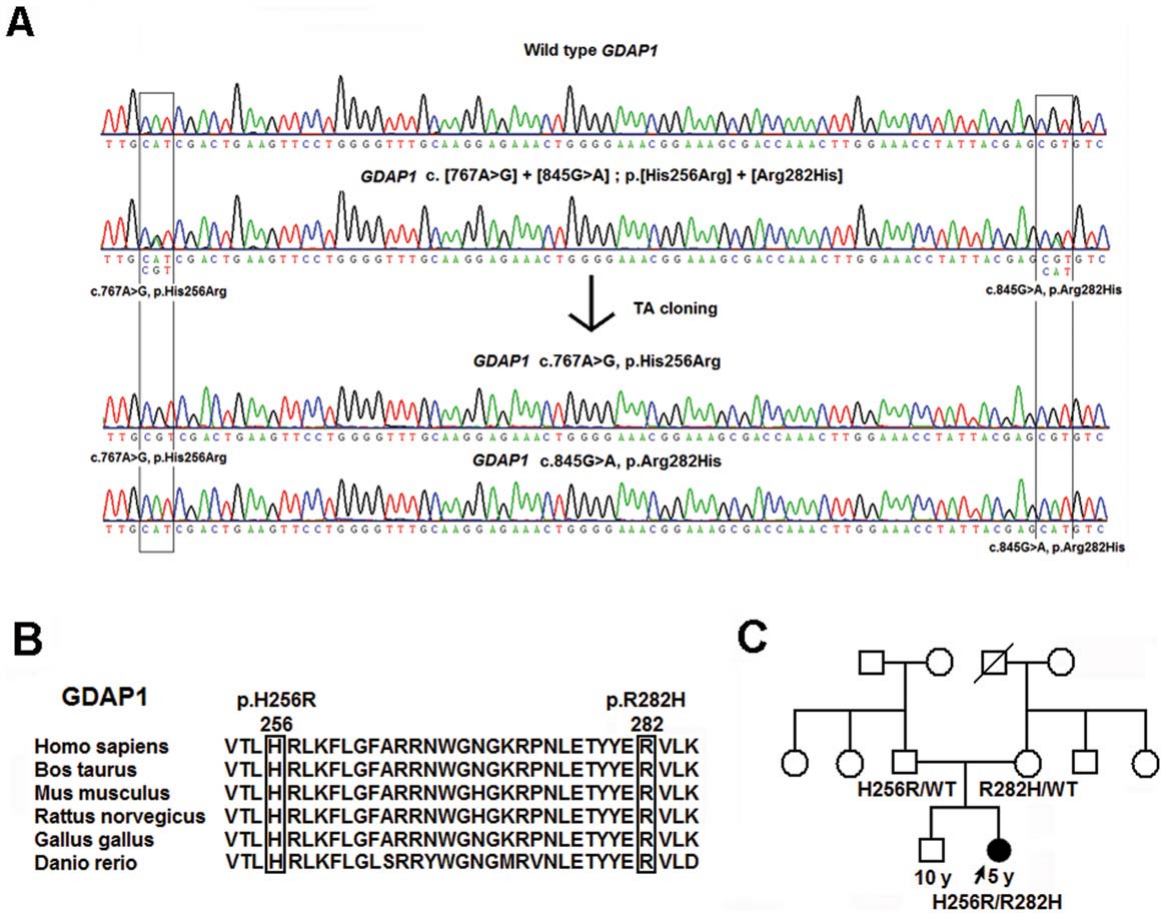
*GDAP1* p.H256 and R282 are evolutionarily conserved, and the p.[H256R]+[R282H] mutations segregate with recessive CMT2. (A) The *GDAP1* compound heterozygous mutations c. [767A>G]+[c.845G>A], which putatively result in p.[H256R]+[R282H], are shown by sequencing TA-subcloned PCR fragments. (B) The evolutionarily conservation of *GDAP1* p.H256 and R282 is shown using a protein sequence alignment of GDAP1 orthologs. (C) The pedigree shows segregation of the *GDAP1* p.[H256R]+[R282H] mutation with autosomal recessive CMT.

**Table 1:** Genetic and clinical information of patients with Charcot-Marie-Tooth diseases type 2 in this study.

Patient, gender	Age (yrs)	Gene	Nucleotide changes	Amino acid change	Age of onset (yrs)	MNCV (m/s, mV)	Family history	CMTNS[45]
*1, M	51	*AARS*	c.211A>T	p.N71Y	30	m38.1, 6.3; u42.1, 6.5; pNR	mother, siblings, son	9
*2, M	34	*HSPB1*	c.490A>G	p.T164A	20	m32.1, 1.8; u34.5, 2.4; pNR	father, sister	24
*3, F	5	*GDAP1*	c. [767A>G]+[845G>A]	p.[H256R]+ [R282H]	< 1	m35.1, 2.3; u48.1, 1.8; pNR	no	13
4, F	26	*NEFL*	c.23C>G	p.P8R	10	m40.8, 0.5; u48.7, 0.6; pNR	father, brother	16
5, M	47	*NEFL*	c.23C>G	p.P8R	10	m38.2, 1.5; u37.8, 1.0; pNR	no	13
6, F	45	*NEFL*	c.64C>T	p.P22S	12	m33, 0.3; u41, 2.4; pNR	father, siblings	12
7, F	43	*NEFL*	c.1186G>A	p.E396K	30	m47.9, 2.6; u43.5, 2.5; p33.5, 0.2	mother, siblings	16
8, M	27	*NEFL*	c.1186G>A	p.E396K	< 1	m41.3, 1.6; u36.8, 5.6; pNR	sister	10
9, F	60	*NEFL*	c.1186G>A	p.E396K	40	m40.3, 7.5; u43.3, 5.4; p27.1, 1.2	no	11
*10, M	20	*MFN2*	c.475-1G>T	p.T159_Q162del	13	m56.5, 9.8; u60.7,12.6; p38.8, 3.4	father, brother	7
*11, M	41	*MFN2*	c.697C>G	p.L233V	12	m65.3, 9; u57.6, 4.6; p44, 6.5	brother	3
12, M	6	*MFN2*	c.1090C>T	p.R364W	2	m37.2, 4; u42.9, 4.5; pNR	no	15
13, M	13	*MFN2*	c.1090C>T	p.R364W	2	m40.2, 0.2; uNR; pNR	no	23
*14, M	32	*MFN2*	c.2230_2231 delinsAT	p.E744M	8	m49, 5.2; u48.2, 3.9; pNR	father	13

Abbreviations: MNCV, motor nerve conduction velocity; yrs, years; m/s, meter per second; mV, millivolt; m, median nerve; u, ulnar nerve; p, peroneal nerve; NR, no response; CMTNS, Charcot-Marie-Tooth Neuropathy Score [38].

*These patients have novel mutations.

Six of the identified mutations were novel, including p.[H256R]+[R282H] in *GDAP1*, p.T164A in *HSP27*, p.N71Y in *AARS*, and c.475-1G>T, p.L233V and p.E744M in *MFN2*. Each of these mutations occurs in an evolutionarily conserved region in the genes and segregates with CMT phenotype within the pedigrees (Figure 1B, 1C, 2B, 2C, 3B, 3C, Figure S2). None of these mutations were found in the 1000 Genomes database (http://browser.1000genomes.org) and 500 control subjects in our population.

**Figure 4:**
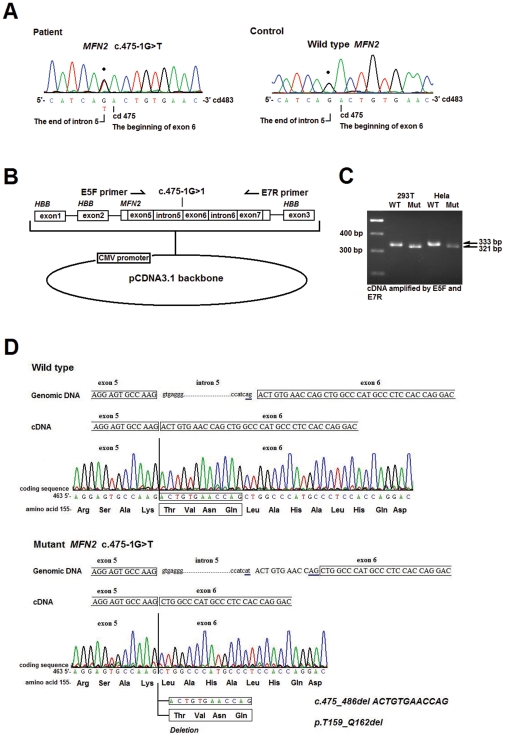
The *MFN2* c.475-1G>T splice acceptor site mutation resulting in a 4-amino acid deletion. (A) The genetic DNA electropherograms of the *MFN2* c.475-1G>T mutation and wild type *MFN2*. (B) The structure of the minigene comprising the *MFN2* genomic sequence from the 3′ end of intron 4 to the 5′ end of intron 7 cloned into intron 2 of the human beta-globulin (*HBB)* gene. The arrows depict the sequences that correspond to the primers that were used in the expression studies. (C) RT-PCR amplification of the minigene constructs (expressed in 293T and HeLa cells) using primers that are specific for *MFN2* exons 5 and 7 (E5F and E7R). The wild type construct (WT) is correctly spliced, whereas the mutant construct (Mut) containing the *MFN2* c.475-1G>T is aberrantly spliced, leading to a 12-bp cDNA fragment deletion. (D) The genomic sequences and cDNA electropherograms of the *MFN2* exon 5 and 6 junction. The wild type, mutant, and cryptic splice acceptor site generated in the mutant allele are underlined. The *MFN2* c.475-1G>T splice acceptor site mutation causes a 12-bp deletion in the cDNA (c.475_486delACTGTGAACCAG) and putatively a deletion of 4 amino acids in the *MFN2* protein (p.T159_Q162del).

The minigene assay showed that the *MFN2* c.475-1G>T mutation, which affects the AG splice acceptor site, results in a deletion of 12 nucleotides in the mRNA (c.475_486delACTGTGAACCAG), corresponding to the loss of 4 amino acid residues in the protein (p.T159_Q162del; Figure 4). Haplotype analysis of the three unrelated patients with the *NEFL* p.E396K mutation revealed no common founder chromosome using seven polymorphic microsatellite markers flanking the *NEFL* gene, indicating that their mutations did not arise from a common ancestor (Figure S1 and Table S2).

The clinical features of the rare *AARS, HSPB1, GDAP1,* and *MFN2* c.475-1G>T mutations are briefly described here. Patient 1 (the proband II:1 in Figure 1C), who carries the *AARS* p.N71Y mutation, presented at age 51 with a slowly progressive weakness and atrophy of the legs that began at the age of 30. He had had normal developmental milestones and finished the military service at age 20. Physical examinations revealed marked atrophy and mild weakness of the muscles in the legs and feet (scoring a 4/5 on the Medical Research Council scale) and mild atrophy and weakness of the intrinsic hand muscles (score 4+/5). This patient also had absent ankle reflexes, diminished other deep tendon reflexes (DTR), and a mildly diminished sensation of all modalities in the distal parts of the extremities without sensory complaints. The proband's mother (I:2), brother (II:4) and son (III:2) had similar clinical manifestations, but their ages of onset varied widely (45, 40 and 11 years, respectively). The proband's two younger sisters (II:3 and II:6) and niece (III:3) denied to have any neurological symptoms; however, neurological examinations uncovered mild atrophy, weakness in the intrinsic foot muscles (score 4+/5) and generalized hyporeflexia in all of them.

Patient 2 (II:3 in Figure 2C) carrying the *HSPB1* p.T164A mutation, which is located in the α-crystallin domain of the heat shock protein beta-1 (HspB1), has experienced a slowly progressive weakness, muscle atrophy and sensory loss in the distal limbs since the age of 20. He had severe sensory loss that resulted in insensitivity to pain and temperature and subsequently suffered repeated injuries with chronic ulcers on the feet since the age of 24. Physical examinations at age 34 revealed a severe atrophy and weakness of the muscles in the legs and in the intrinsic muscles of the hands and feet (score 0-1/5), absent knee and ankle reflexes and decreased upper-limb DTRs, and a severely diminished sensation of all modalities in the regions below the elbows and knees. His father (I:1) had a similar clinical presentation at the age of 29 with frequent trauma and burn injuries to the feet since age 32. His walking had been restricted to the use of a walker after age 60, and he became wheelchair-bound at age 66. He died of a pneumonia at age 76. The proband's eldest sister (II:1) has had similar but milder clinical manifestations since the age of 32. Physical examinations at age 40 revealed a marked atrophy and weakness of the muscles in the legs and feet (score 2−3/5), mild atrophy and weakness of the intrinsic hand muscles (score 4−4+/5), absent ankle and knee reflexes and preserved DTRs of the upper-limbs, and mildly diminished sensation of all modalities in the distal parts of four limbs.

Patient 3 (Figure 3B), who carried the *GDAP1* p.[H256R]+[R282H] mutation, had a delayed onset of ambulation at 18 months of age and an unsteady gait after 2 years of age. Physical examinations at age 5 revealed a mild atrophy and weakness of the intrinsic muscles of the feet (score 2/5), impaired dorsiflexion of the feet (score 2−3/5), generalized areflexia, and mildly diminished sensation of all modalities in regions distal to the wrists and ankles, despite a lack of sensory complaints. She did not have any relatives with similar presentations.

Patient 10 (Figure S2A), who carried the *MFN2* c.475-1G>T mutation, presented at age 20 with a slowly progressive weakness and atrophy of the distal lower limbs with occasional numbness since the age of 13. Physical examinations revealed pes cavus, marked atrophy and mild weakness of the muscles in the legs and feet (score 4−4+/5) but sparing the upper-limb muscles, absent ankle reflexes and generalized decreases in other DTRs, and mildly diminished sensation of all modalities in the distal parts of all extremities without sensory complaints. He did not have any features to suggest a brain involvement. His father and younger brother had a similar clinical manifestation with an onset at approximately 10 years of age.

## Discussion

In this study, we conducted an extensive survey for the mutations in the *MFN2, RAB7, TRPV4, GARS, NEFL, HSPB1, MPZ, GDAP1, HSPB8, DNM2, AARS* and *YARS* genes in 36 unrelated patients with CMT2 and identified 10 different mutations in 14 patients (10/36; 38.9% of our cohort). In the study cohort, the frequency of *NEFL* mutations (6/36; 16.7%) was unexpectedly high, and the frequency of *MFN2* mutations (5/36; 13.9%) was consistent with other reports (11–24.2%) in previous studies [19]–[22]. The high frequency of *NEFL* mutations was not the result of a founder effect (Table S1). Moreover, the pathogenicity of the three rare mutations in CMT2 (i.e., the *AARS* p.N71Y, *HSP27* p.T164A, and *GDAP1* p.[H256R]+[R282H]) was demonstrated because these mutations segregated with CMT in the families, involved highly conserved amino acids, and were absent on the 1000 control chromosomes. We also used the minigene assay to demonstrate that the *MFN2* splice acceptor site mutation c.475-1G>T may cause abnormal mRNA splicing and result in a 4-amino acid deletion (p.T159_Q162del). Since the study cohort was selected from a consecutive series of 251 CMT pedigrees and we did not find any *NEFL* or *GDAP1* mutations among the index patients (data not shown), the mutation frequencies for CMT in our population were 2.4% (6/251) for *NEFL*, 2.0% (5/251) for *MFN2*, and 0.4% (1/251) for each *of AARS*, *HSPB1* and *GDAP1*.

Patient 1 carries a rare mutation in the *AARS* gene, which codes for alanyl-tRNA synthetase (AlaRS), an enzyme that is essential for protein synthesis by catalyzing the aminoacylation of tRNA^Ala^ molecules with alanine. This process loads the tRNA^Ala^ with alanine. A single mutation in the helical domain of AlaRS (p.R329H) was recently described in two families with autosomal dominant axonal CMT (CMT2N) [16]. The *AARS* p.N71Y mutation, which segregates with disease in the family, is located in the aminoacylation domain of AlaRS and may compromise the aminoacylation reaction. Indeed, recent functional analyses (Dr. Anthony Antonellis, personal communication, manuscript in preparation) demonstrated that N71Y AlaRS severely impairs enzyme activity *in vitro* and *in vivo*. The phenotype in our patients with this *AARS* mutation was similar to that reported previously with the *AARS* p.R329H mutation. All of the patients were ambulatory and had pure axonal neuropathy with a variable age of onset (ranging from 11–45 years of age). AlaRS is the third aminoacyl-tRNA synthetase that has been found to be involved in CMT. CMT can also result from defects in glycyl-tRNA synthetase, tyrosyl-tRNA synthetase, or lysyl-tRNA synthetase, although these are rare [6], [24], [29]. Why mutations in these ubiquitously expressed genes result solely in neuropathy remains unknown. This study helps confirm the involvement of *AARS* mutations in CMT and further demonstrates the role of aminoacyl-tRNA synthetase in its pathogenesis.

Patient 2 carries a rare, novel mutation in the *HSPB1* gene, which encodes the small heat shock protein HspB1, a member of the chaperone family. Small heat shock proteins (sHSPs) play an important role in cellular defense during stress by binding misfolded or denatured proteins to protect them from irreversible aggregation [30]. They are ubiquitously expressed and are characterized by the presence of a conserved α-crystallin domain. The α-crystallin domain is essential for dimerization and oligomerization of HspB1 [31], which are required for properly functioning of HspB1 [30]. Only a few *HSPB1* mutations have previously been identified in families with CMT2F and families with distal hereditary motor neuropathy (HMN2B), and most of them are located in the α-crystallin domain [8], [32]. The *HSPB1* p.T164A mutation in Patent 2 is also located in the α-crystallin domain and may influence the HspB1 function. The definitive patho-mechanisms of *HSPB1* mutations remain elusive and can't be fully explained by alteration of chaperone function. One recent *in vitro* study showed that *HSPB1* R127W and S135F mutations significantly increased the chaperone activity of HspB1, R136W mutation increased the chaperone activity slightly, and T151L and P182L mutations did not change the chaperone activity [33]. In addition to chaperone function, HspB1 has also been implicated in regulating apoptotic pathways [34], enhancing oxidative defense [35], and modulating cytoskeleton dynamics [36]. The phenotypes of the *HSPB1* mutations that have been reported to date are motor-predominant neuropathy either with or without mild sensory involvement. However, Patient 2 had sensorimotor polyneuropathy with a severe sensory loss that resulted in repeated foot injuries. This feature broadens the clinical spectrum of *HSPB1* neuropathy.

Patient 3 carries a rare, novel compound heterozygous mutation in the *GDAP1* gene, which encodes ganglioside-induced differentiation-associated protein 1 (GDAP1). GDAP1 regulates mitochondrial dynamics by inducing mitochondrial fission [37], which helps regulate mitochondrial function by controlling mitochondrial morphology. *GDAP1* mutations have previously been reported in patients with autosomal dominant axonal CMT (CMT2K) [11], recessive demyelinating CMT (CMT4A) [11], recessive intermediate CMT (CMTRIA) [38], and recessive axonal CMT with vocal cord paresis [12]. The clinical manifestations of Patient 3 were similar to those reported previously in patients with *GDAP1*-related recessive axonal CMT, who presented with severe sensorimotor polyneuropathy and a disease onset under the age of 2. The *GDAP1* p. [H256R]+[R282H] mutations are located in the GST-C (glutathione-S-transferases, C-terminal region) domain. Although a previous study showed that GDAP1 did not have functional GST activity [39], the GST domains are important in GDAP1 function, as many *GDAP1* mutations that are linked to CMT involve the GST domains [40].

Patient 10 carries a rare, novel *MFN2* acceptor splice site mutation, that alters the consensus acceptor splice site of intron 5 from AG to AT and affects *MFN2* splicing and expression. *In vitro* splicing assays are valuable for evaluating the influence of splicing mutations. *MFN2* c.1392+2T>C, a mutation that affects the donor splice site of intron 13, was recently identified in a family with severe CMT2 and fatal encephalopathy [28]. Functional analysis revealed that this splice site mutation might lead to four aberrant mRNA transcripts, which are predicted to result in two different truncating frameshift mutations with two approximate 260-amino acid deletions, one mutation with a 15-amino acid insertion and one mutation with a 4-amino acid deletion [28]. The *MFN2* c.475-1G>T, which is carried by Patient 10, was found to cause a deletion of 4 amino acids (p.T159_Q162del), and this may explain his relatively mild polyneuropathy.

Haplotype analysis revealed that Patients 7, 8 and 9, each of whom carry the *NEFL* p.E396K mutation, did not have a common founder. This suggests that E396 is a hot-spot for this spontaneous mutation in the *NEFL* gene. This is supported by the fact that the *NEFL* p.E396K mutation has also been reported in German, Korean and Japanese patients with CMT despite the relative rarity of *NEFL* mutations [41]–[43]. R364 in the mitofusin 2 protein may also be a hot-spot for *MFN2* mutations. Patients 12 and 13 both carry the *de novo MFN2* p.R364W mutation and both had severe and early onset CMT2. Among various populations, more than 12 patients carrying the *MFN2* p.R364W or p.R364P mutation have been reported to have severe early onset CMT2 [19]–[22], which is obviously disadvantageous for reproduction. Methylation and deamination of cytosine within a CpG dinucleotide, which results in transitions of cytosine to thymine [44], may play an important role in the recurrent mutations of *NEFL* p.E396K (c.1186G>A, which causes ggcGAG>ggcAAG), *MFN2* R364W (c.1090C>T, which causes CGG>TGG), and *MFN2* R364P (c.1091G>A, which causes CGG>TAG), as they each occur at the cytosine residue of a CpG dinucleotide in the sense or antisense strand.

Our survey of mutations previously implicated in CMT2 revealed that the genetic causes of the disease in approximately 60% of patients in our cohort remain unidentified. Although a few additional, rare CMT2 genes have not been screened, these genetically unassigned patients likely reflect additional genetic heterogeneity within the CMT2 syndrome.

The limitation of this study is that we had difficulty recruiting more family members of the CMT2 pedigrees with the novel mutations, and most of these pedigrees are small for segregation analysis to firmly support the pathogenic roles of these mutations. However, the pathogenic role of these novel disease-causing mutations are still implicated by the involvement of highly conserved amino acids and their absence in 1000 control chromosomes.

In conclusion, this study demonstrated the spectrum of CMT2 mutations in ethnic Chinese patients and expands the list of CMT2-associated mutations. The importance of mutations in the *AARS* and *HSPB1* genes in the pathogenesis of axonal CMT was further highlighted. The frequency of the *NEFL* mutation in our population was unexpectedly high. Mutations in the *NEFL* and *MFN2* genes should be ascertained first during genetic testing for CMT2 in patients of Chinese origin.

## Supporting Information

Figure S1 **Three unrelated CMT2E pedigrees carrying the** ***NEFL*** **E396K mutation.** Patients 7 (A), 8 (B) and 9 (C) are indicated with arrows. Asterisks (*) depicting the individuals who received genetic screening of *NEFL* and haplotype analysis.
(DOC)

Figure S2 **Three CMT2A pedigrees carrying novel** ***MFN2*** **mutations at phylogenetically conserved sites.** The novel *MFN2* mutations in the pedigrees of Patients 10 (A), 11 (B) and 14 (C) are located in the phylogenetically conserved regions. Shown are the electropherogams, pedigrees and nucleotide sequence alignments of *MFN2* orthologs, or protein sequence alignments of mitofusin 2 orthologs.
(DOC)

Table S1 The mode of inheritance and electrophysiological features of the 36 unrelated patients with Charcot-Marie-Tooth disease type 2.
(DOCX)

Table S2 The haplotypes linked to the *NEFL* p.E396K mutation in the three unrelated CMT2E patients.
(DOC)
