# Correction: The Mutational Spectrum in a Cohort of Charcot-Marie-Tooth Disease Type 2 among the Han Chinese in Taiwan

**DOI:** 10.1371/annotation/237439db-35ae-4884-9ee7-2ae19872a581

**Published:** 2012-01-23

**Authors:** Kon-Ping Lin, Bing-Wen Soong, Chih-Chao Yang, Li-Wen Huang, Ming-Hong Chang, I-Hui Lee, Anthony Antonellis, Yi-Chung Lee

There was a spelling error in the name of the seventh author. The correct spelling is Anthony Antonellis. 

