# Supplementary material for: The Mutational Spectrum in a Cohort of Charcot-Marie-Tooth Disease Type 2 among the Han Chinese in Taiwan
Source: PLoS One. 2011 Dec 19;6(12):e29393. doi: 10.1371/journal.pone.0029393 (PMC3242783; doi:10.1371/journal.pone.0029393)
Supplement: Figure S1 — Three unrelated CMT2E pedigrees carrying the NEFL E396K mutation. Patients 7 (A), 8 (B) and 9 (C) are indicated with arrows. Asterisks (*) depicting the individuals who received genetic screening of NEFL and haplotype analysis. (DOC) [file pone.0029393.s001.doc]

**Figure S1 Three unrelated CMT2E pedigrees carry the *NEFL* E396K mutation.** Patients 7 (A), 8 (B) and 9 (C) are indicated with arrows. Asterisks (*) depicting the individuals who received genetic screening of *NEFL* and haplotype analysis.

**
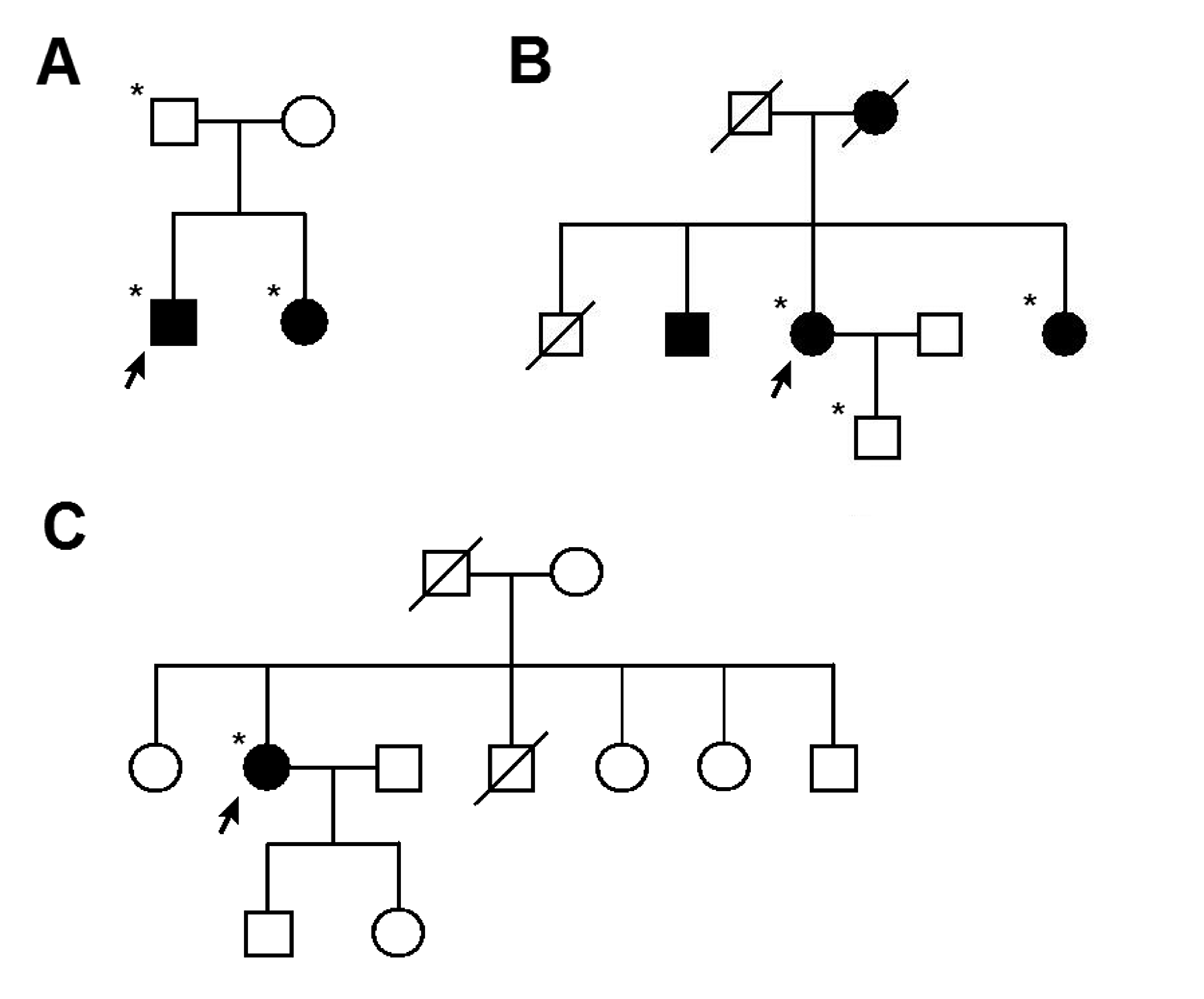
**
