# Supplementary material for: The Mutational Spectrum in a Cohort of Charcot-Marie-Tooth Disease Type 2 among the Han Chinese in Taiwan
Source: PLoS One. 2011 Dec 19;6(12):e29393. doi: 10.1371/journal.pone.0029393 (PMC3242783; doi:10.1371/journal.pone.0029393)
Supplement: Figure S2 — Three CMT2A pedigrees carrying novel MFN2 mutations at phylogenetically conserved sites. The novel MFN2 mutations in the pedigrees of Patients 10 (A), 11 (B) and 14 (C) are located in the phylogenetically conserved regions. Shown are the electropherogams, pedigrees and nucleotide sequence alignments of MFN2 orthologs, or protein sequence alignments of mitofusin 2 orthologs. (DOC) [file pone.0029393.s002.doc]

**Figure S2 Three CMT2A pedigrees carry novel *MFN2* mutations in phylogenetically conserved sites.** The novel *MFN2* mutations in the pedigrees of Patients 10 (A), 11 (B) and 14 (C) are located in the phylogenetically conserved regions. Shown are the electropherogams, pedigrees and nucleotide sequence alignments of *MFN2* orthologs, or protein sequence alignments of mitofusin 2 orthologs.

**
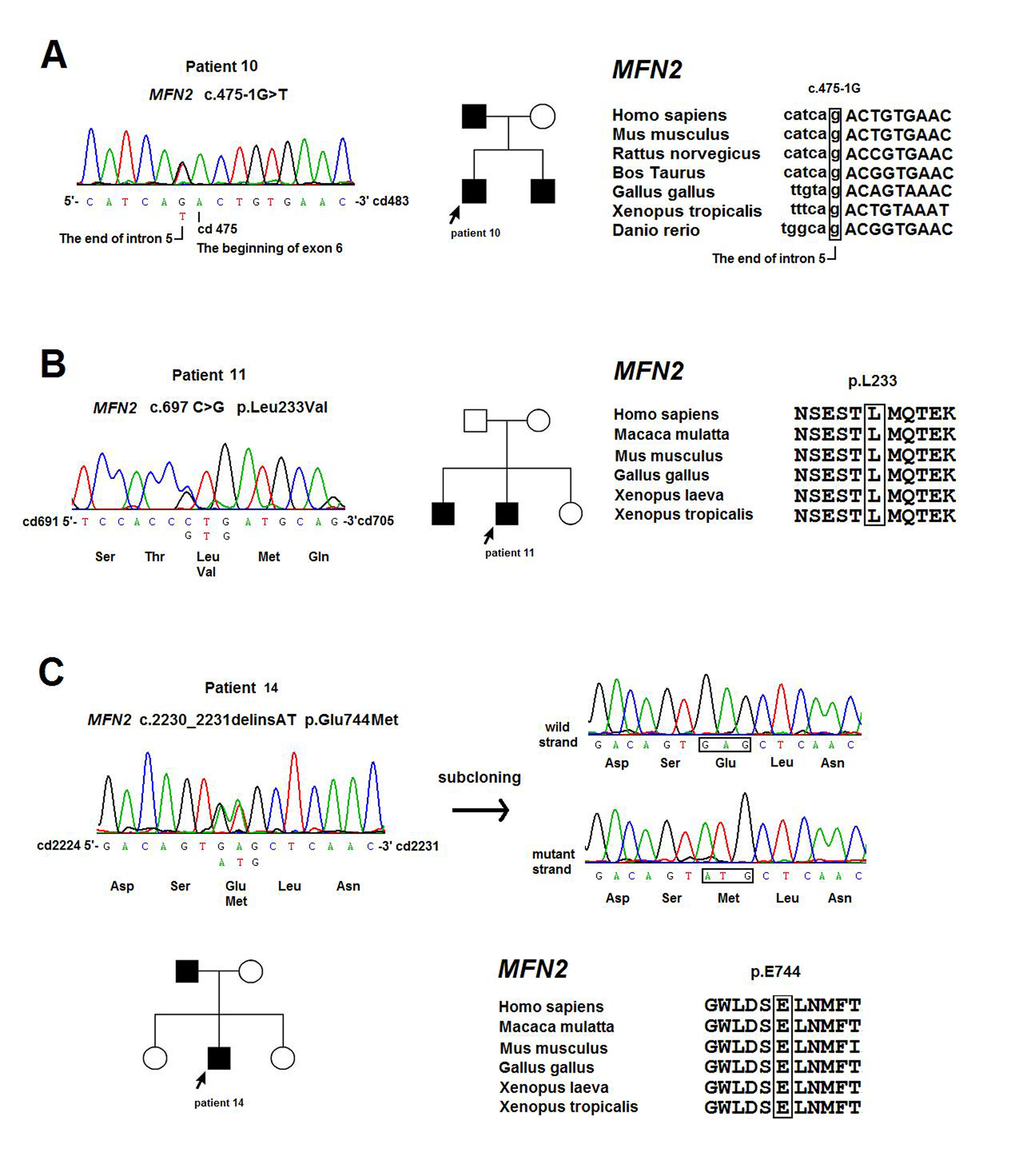
**
