# Supplementary material for: The Mutational Spectrum in a Cohort of Charcot-Marie-Tooth Disease Type 2 among the Han Chinese in Taiwan
Source: PLoS One. 2011 Dec 19;6(12):e29393. doi: 10.1371/journal.pone.0029393 (PMC3242783; doi:10.1371/journal.pone.0029393)
Supplement: Table S1 — The mode of inheritance and electrophysiological features of the 36 unrelated patients with Charcot-Marie-Tooth disease type 2. (DOCX) [file pone.0029393.s003.docx]

| **Table S1** The mode of inheritance and electrophysiological data of the 36 unrelated patients with Charcot-Marie-Tooth disease type 2 | | | | | | | | | | | | | |
| --- | --- | --- | --- | --- | --- | --- | --- | --- | --- | --- | --- | --- | --- |
| Patient | Gender | Age at NCS (yrs) | Mode of inheritance | Motor NCS | | | | Sensory NCS | | | | | |
|  |  |  |  | Median nerve | | Peroneal nerve | | Median nerve | | Ulnar nerve | | Sural nerve | |
|  |  |  |  | NCV, m/s | CMAP, mV | NCV, m/s | CMAP, mV | DL,  ms | SNAP, uV | DL, ms | SNAP, uV | DL, ms | SNAP, uV |
| Normal value^1^🡪 | |  |  | ≧51.9^2^ | ≧6.4^2^ | ≧42^2^ | ≧2.1^2^ | ≦2.9^3^ | ≧17^2^ | ≦3.0^3^ | ≧17^2^ | ≦3.3^3^ | ≧12^2^ |
| 1^4^ | M | 51 | AD | 38.1 | 6.3 | NR | NR | NR | NR | NR | NR | NR | NR |
| 2^4^ | F | 5 | AS | 35.1 | 2.3 | NR | NR | 2.7 | 7.4 | 1.7 | 7.8 | NR | NR |
| 3^4^ | M | 34 | AD | 32.1 | 1.8 | NR | NR | NR | NR | NR | NR | NR | NR |
| 4^4^ | F | 26 | AD | 40.8 | 0.5 | NR | NR | NR | NR | NR | NR | NR | NR |
| 5^4^ | M | 47 | AS | 38.2 | 1.5 | NR | NR | NR | NR | NR | NR | NR | NR |
| 6^4^ | F | 45 | AD | 33 | 0.3 | NR | NR | NR | NR | NR | NR | NR | NR |
| 7^4^ | F | 43 | AD | 47.9 | 2.6 | 33.5 | 0.2 | NR | NR | NR | NR | NR | NR |
| 8^4^ | M | 27 | AR | 41.3 | 1.6 | NR | NR | NR | NR | NR | NR | NR | NR |
| 9^4^ | F | 60 | AD | 40.3 | 7.5 | 27.1 | 1.2 | NR | NR | 4.4 | 1.2 | NR | NR |
| 10^4^ | M | 20 | AD | 56.5 | 9.8 | 38.8 | 3.4 | 3.3 | 9 | 2.9 | 9 | NR | NR |
| 11^4^ | M | 41 | AR | 65.3 | 9 | 44 | 6.5 | 2.5 | 17 | 2.6 | 11.6 | 3.1 | 8.8 |
| 12^4^ | M | 6 | AS | 37.2 | 4 | NR | NR | NR | NR | NR | NR | NR | NR |
| 13^4^ | M | 13 | AS | 40.2 | 0.2 | NR | NR | NR | NR | NR | NR | NR | NR |
| 14^4^ | M | 32 | AD | 49 | 5.2 | NR | NR | NR | NR | NR | NR | NR | NR |
| 15 | F | 46 | AD | 41.1 | 7.3 | 39 | 0.5 | 3.9 | 10 | 2.6 | 7 | NR | NR |
| 16 | F | 14 | AD | 44.2 | 0.3 | NR | NR | NR | NR | NR | NR | NR | NR |
| 17 | M | 33 | AD | 44.7 | 2.2 | NR | NR | NR | NR | NR | NR | NR | NR |
| 18 | M | 50 | AD | NR | NR | 45 | 0.5 | 2 | 11.4 | 2.3 | 12.2 | 2.6 | 8.5 |
| 19 | M | 51 | AD | 46.6 | 5.5 | 30.7 | 0.3 | 3.1 | 16.8 | 2.9 | 14.4 | NR | NR |
| 20 | F | 21 | AS | 56.1 | 4 | NR | NR | NR | NR | NR | NR | NR | NR |
| 21 | M | 62 | AR | 60.2 | 4.5 | 42.1 | 1 | 2.44 | 9 | 3.74 | 7.2 | NR | NR |
| 22 | M | 25 | AS | 38.5 | 6.2 | NR | NR | 3.92 | 8.6 | NR | NR | NR | NR |
| 23 | M | 59 | AR | 43.6 | 2.4 | 27.6 | 0.4 | NR | NR | NR | NR | NR | NR |
| 24 | M | 57 | AD | 56 | 9 | 35.3 | 1.3 | NR | NR | NR | NR | NR | NR |
| 25 | F | 38 | AS | 49.4 | 5.6 | NR | NR | 3.5 | 4.4 | 4 | 6.8 | NR | NR |
| 26 | M | 42 | AR | 55.2 | 7.4 | 33.8 | 0.2 | 3.12 | 8.8 | 3.1 | 7.6 | NR | NR |
| 27 | M | 30 | AD | 48 | 1.2 | 38.3 | 0.1 | 3.5 | 7.6 | 3.6 | 11.6 | 3.7 | 8 |
| 28 | F | 36 | AR | 60.3 | 13.3 | NR | NR | 2.8 | 26 | 2.7 | 16 | 2.9 | 8.4 |
| 29 | M | 55 | AR | 41.8 | 0.6 | NR | NR | 2.6 | 17 | 2.4 | 9.5 | 2.9 | 5 |
| 30 | F | 35 | AD | 56.7 | 5.7 | 48.5 | 3.1 | 2.8 | 8.4 | 2.7 | 8.4 | NR | NR |
| 31 | M | 31 | AD | NR | NR | NR | NR | NR | NR | NR | NR | NR | NR |
| 32 | F | 22 | AS | 61.4 | 8.8 | NR | NR | 4.2 | 6.8 | 3.2 | 6.8 | NR | NR |
| 33 | M | 18 | AD | 39.8 | 7.4 | 31.4 | 0.4 | 3.8 | 7.3 | NR | NR | NR | NR |
| 34 | M | 62 | AD | 44.2 | 6.1 | NR | NR | 3.4 | 6.8 | NR | NR | NR | NR |
| 35 | M | 22 | AS | 60.7 | 7.6 | 50.7 | 1.5 | 3.4 | 18.7 | 2.9 | 13.2 | 2.7 | 8.3 |
| 36 | M | 36 | AD | 56.8 | 1.9 | 48.5 | 2 | 2.4 | 10.1 | 2.2 | 10.4 | 3.2 | 4.1 |

NCS: nerve conduction study; NCV: nerve conduction velocity; CMAP: compound muscle action potential; SNAP: sensory nerve action potential; DL: distal latency; yrs: years; m/s: meter per second; mV: millivolt; ms: millisecond; uV: microvolt; AD: autosomal dominant; AR: autosomal recessive; AS: apparently sporadic; NR: no response.

^1^The normal values in 100 nerves from 50 Han Chinese individuals, 20 to 64 years of age (average, 38), with no apparent disease of the peripheral nerves.

^2^Lower limits of normal, calculated as the mean – 2 standard deviation (SD).

^3^Upper limits of normal, calculated as the mean + 2 SD.

^4^The genetic and clinical information of these patients were also listed in Table 1.
