# Supplementary material for: The Mutational Spectrum in a Cohort of Charcot-Marie-Tooth Disease Type 2 among the Han Chinese in Taiwan
Source: PLoS One. 2011 Dec 19;6(12):e29393. doi: 10.1371/journal.pone.0029393 (PMC3242783; doi:10.1371/journal.pone.0029393)
Supplement: Table S2 — The haplotypes linked to the NEFL p.E396K mutation in the three unrelated CMT2E patients. (DOC) [file pone.0029393.s004.doc]

**Table S2** The haplotypes linked to the *NEFL* p.E396K mutation in the three unrelated CMT2E patients

| Locus | D8S1734 | | D8S481 | | D8S1989 | | *NEFL* | | D8S1739 | | D8S1771 | | D8S382 | | D8S1839 |
| --- | --- | --- | --- | --- | --- | --- | --- | --- | --- | --- | --- | --- | --- | --- | --- |
| ^a^Distance | | 1.5 KcM | | 0.3 KcM | | 0.21 KcM | | 0.13 KcM | | 0.46 KcM | | 0.55 KcM | | 1.45 KcM | |
| Patient 7 | ^b^2/3 | | 4 | | 2 | | p.E396K | | 2 | | 1 | | 2 | | 3 |
| Patient 8 | 3 | | 4 | | 2 | | p.E396K | | 3 | | 2 | | 2 | | 2 |
| Patient 9 | 1/3 | | 1/4 | | 3/4 | | p.E396K | | 3 | | 3 | | 1 | | 2/5 |

Abbreviation: *KcM* = Kosambi centimorgan.

^a^Sex-averaged genetic distances of the markers are shown in the top row.

^b^Alleles with an unknown phase are separated with a slash.
